# Pre-Stimulus Power but Not Phase Predicts Prefrontal Cortical Excitability in TMS-EEG

**DOI:** 10.3390/bios13020220

**Published:** 2023-02-03

**Authors:** Mohsen Poorganji, Reza Zomorrodi, Christoph Zrenner, Aiyush Bansal, Colin Hawco, Aron T. Hill, Itay Hadas, Tarek K. Rajji, Robert Chen, Brigitte Zrenner, Daphne Voineskos, Daniel M. Blumberger, Zafiris J. Daskalakis

**Affiliations:** 1 Temerty Centre for Therapeutic Brain Intervention, Centre for Addiction and Mental Health, Toronto, ON M6J 1H4, Canada; 2 Institute of Medical Science, University of Toronto, Toronto, ON M5S 1A8, Canada; 3 Institute for Biomedical Engineering, University of Toronto, Toronto, ON M5S 3G9, Canada; 4 Department of Psychiatry, University of Toronto, Toronto, ON M5T 1R8, Canada; 5 Cognitive Neuroscience Unit, School of Psychology, Deakin University, Melbourne, VIC 3125, Australia; 6 Department of Psychiatry, School of Medicine, University of California San Diego, La Jolla, CA 92093-0603, USA; 7 Toronto Dementia Research Alliance, University of Toronto, Toronto, ON M5S 1A8, Canada; 8 Division of Neurology, Department of Medicine, University of Toronto, Toronto, ON M5S 1A1, Canada; 9 Krembil Research Institute, University Health Network, Toronto, ON M5T 0S8, Canada

**Keywords:** TMS-EEG, cortical oscillations, brain state, phase before TMS, EEG power before TMS

## Abstract

The cortical response to transcranial magnetic stimulation (TMS) has notable inter-trial variability. One source of this variability can be the influence of the phase and power of pre-stimulus neuronal oscillations on single-trial TMS responses. Here, we investigate the effect of brain oscillatory activity on TMS response in 49 distinct healthy participants (64 datasets) who had received single-pulse TMS over the left dorsolateral prefrontal cortex. Across all frequency bands of theta (4–7 Hz), alpha (8–13 Hz), and beta (14–30 Hz), there was no significant effect of pre-TMS phase on single-trial cortical evoked activity. After high-powered oscillations, whether followed by a TMS pulse or not, the subsequent activity was larger than after low-powered oscillations. We further defined a measure, *corrected_effect*, to enable us to investigate brain responses to the TMS pulse disentangled from the power of ongoing (spontaneous) oscillations. The *corrected_effect* was significantly different from zero (meaningful added effect of TMS) only in theta and beta bands. Our results suggest that brain state prior to stimulation might play some role in shaping the subsequent TMS-EEG response. Specifically, our findings indicate that the power of ongoing oscillatory activity, but not phase, can influence brain responses to TMS. Aligning the TMS pulse with specific power thresholds of an EEG signal might therefore reduce variability in neurophysiological measurements and also has the potential to facilitate more robust therapeutic effects of stimulation.

## 1. Introduction

Transcranial magnetic stimulation (TMS) non-invasively stimulates the brain by delivering magnetic pulses over the scalp [1]. TMS has shown promise as a targeted therapeutic intervention in both psychiatric and neurological disorders [2] as well as a useful neurophysiological tool, especially when combined with electromyography (TMS-EMG) or electroencephalography (TMS-EEG) [3,4,5]. Therapeutic TMS protocols, including repetitive TMS (rTMS) and intermittent theta-burst stimulation (iTBS), have been established as promising treatments for major depressive disorder (MDD) [6,7]; however, remission rates remain limited to approximately 16% [8]. Adjusting TMS parameters such as intensity, number of pulses, and stimulation target site have been linked to better outcomes and are ways to individualize treatment [9]. Therefore, there is significant potential for optimizing existing TMS protocols to maximize neurophysiological engagement and treatment efficacy. 

TMS is conventionally delivered without synchronizing stimulation pulses to the phase of ongoing brain oscillations, despite potentially important differences in the neurophysiological response to TMS depending on the underlying state of the cortex [10,11]. Although it is not possible to access the true state of the brain, EEG can provide insight into the spontaneous oscillatory activity of neuronal populations [12]. Instantaneous oscillatory phase and amplitude can be considered as prominent characteristics of brain state [13,14]. Zrenner et al. (2018) examined the effect of neuronal oscillations on corticospinal excitability, measured by the motor-evoked potential (MEP) response [15]. When compared to delivering the pulses at random, without considering the ongoing spontaneous oscillatory brain dynamics, a higher long-term potentiation-like effect of repetitive transcranial magnetic stimulation (rTMS) over the motor cortex was found when the pulses were synchronized with the negative peak of sensorimotor mu-rhythm [15]. In a further study of patients with MDD, a single session of alpha-synchronized rTMS over the dorsolateral prefrontal cortex (DLPFC) resulted in a specific decrease in resting state alpha activity and increases in TMS-induced beta oscillations over fronto-central electrodes [16]. However, there was no observed difference in TMS-evoked potentials (TEP—the average of all single-trial responses and their components) before and after alpha-synchronized stimulation [16]. Desideri et al. (2018) also reported delivering rTMS over the motor cortex at different phases of mu-rhythm with no effect on TMS-EEG [17]. Inter-stimulus interval also represents a source of variability, as evidenced by a recent investigation which failed to demonstrate a significant effect of the phase of the mu-rhythm on MEP amplitude when single-pulse TMS was applied over the motor cortex [18]. The significant modulatory effect of a phase of EMG beta [19] and the power of EEG alpha [20] oscillations on MEP amplitude was also demonstrated by previous studies.

Although TMS-EEG is now being used as an investigational tool to develop biomarkers to increase the efficacy of neuromodulation-based treatments in psychiatry [21], there has been little research into the difference at the level of single-trial TMS-EEG response (in individual trials (pulses) rather than average of all trials). We performed a retrospective analysis on TMS-EEG data of healthy participants recorded from DLPFC. Here, we are investigating the possible modulatory effect of different pre-TMS phases and power conditions on the magnitude of single-trial cortical responses to TMS. We assess the reactivity of the cortex to stimulation by the previously established measure of cortical-evoked activity (CEA) [22], defined as the area under the curve of the rectified waveform of the TMS-EEG response in the time window of interest for a channel of interest. CEA was previously shown to reflect the total neural activity resulting from a TMS pulse [22,23]. We investigated the effect of the phase and power of ongoing brain oscillations prior to the TMS pulse on the single-trial TMS response, recorded with EEG. We hypothesized that the different phases of EEG signal in the theta, alpha, and beta frequency bands before the TMS pulse would result in significantly different amplitudes for single-trial responses compared to stimulation at a random phase. We also hypothesized that there would be a significant association between the power of the pre-TMS EEG signal and the amplitude of the single-trial TMS response. Understanding the interaction between the state of the brain at the time of TMS and how the brain subsequently responds, as recorded via EEG, could lead to a better understanding of the response variability in TMS-EEG studies and may help us to develop more effective and individualized therapeutic interventions. 

## 2. Methods

### 2.1. Participants

A secondary analysis was performed on a total of 64 EEG recordings from 49 distinct healthy participants. These recordings were taken from two different studies: 34 recordings were taken from a study investigating the auditory effect of TMS-EEG [24], 30 recordings were taken from a second study investigating the effect of auditory masking on TMS-EEG (not published yet) in 15 participants (two sessions per participant). The two blocks included conventional TMS-EEG and delivered the pulses while playing white noise at the maximum level that was tolerable to the participant with a thin layer of foam placed between the underside of the coil and the scalp. The participants (31 female) were right-handed (assessed by Edinburgh Handedness Inventory), aged between 19 and 54, could communicate effectively in English, and were screened for exclusion criteria (no history of neurological disorder, family history of schizophrenia, psychotic disorders, and psychiatric disorders (Diagnostic and Statistical Manual of Mental Disorders, 5th edition (DSM-V) Axis I disorder)). The study protocols of the datasets used in this study were approved by the Centre for Addiction and Mental Health ethics committee. The participants had provided their written consent.

### 2.2. Transcranial Magnetic Stimulation

Magnetic pulses (monophasic) were delivered via a figure-of-eight coil connected to two Magstim-200 stimulators (Magstim Company Ltd., Whitland, UK). To target left DLPFC, an online MRI-neuro-navigated system (Brainsight Neuronavigation) was used with MNI coordinates of x: −35, y: 45, z: 38 using each participant’s T1 anatomical MRI [25]. The MRI was acquired prior to the TMS-EEG session. In total, 100 single-pulses (SP) were delivered with the inter-stimulus interval of five seconds and the intensity at which the magnetic pulse was applied to the left motor cortex would induce an MEP amplitude of ~1 mV peak-to-peak in 10 consecutive trials [2]. The target muscle for determination of the 1 mV peak-to-peak amplitude of MEP was the abductor pollicis brevis of the right hand. One-millivolt peak-to-peak amplitude determination was conducted after the EEG cap was put on the participant’s head. The pulses over DLPFC were delivered with the coil positioned at 45° in relation to the mid-sagittal line.

### 2.3. Electroencephalography

EEG was recorded using a 64 channel (AgCl ring electrode) cap connected to a SynAmps2 amplifier controlled by Neuroscan software (Compumedics Neuroscan). The arrangement of the electrodes was based on the international 10–20 system. The data were recorded with a sampling rate of 20 kHz and were then filtered using DC and low-pass filters (3500 Hz). Throughout the session, the participants were reminded to have their eyes fixated on a cross. Electrode impedances were kept below 5 kOhm. The first 34 TMS-EEG recordings and the 15 datasets of the second recording were performed with the conventional method of TMS-EEG without masking the sensory co-activation. The other 15 datasets in the second recoding were performed with the state-of-the-art method of sensory co-activation by playing white noise for the participants through earphones, covering their ears with earmuffs, and attaching a layer of foam to the coil. 

### 2.4. EEG Pre-Processing

TMS-EEG data were pre-processed using EEGLAB [26], TESA [27], and FieldTrip [28] MATLAB toolboxes (R2018b). In addition, custom scripts developed in our laboratory were used to process the data. The steps taken were based on previous publications by our group [29]. Before epoching the data around the TMS pulse (±1 s), noisy electrodes, or any electrodes with bridging were detected and removed. Data were removed from −2 ms to 20 ms around the TMS pulse (TMS pulse artifact) and linear interpolation was applied, after which noisy trials were deleted. After baseline correction (−500 to −200 ms) and average referencing, the data were down-sampled to 1 kHz. The first round of independent component analysis (ICA) was then applied to remove large muscle artifacts. Next, data were bandpass-filtered (1–100 Hz) and a second round of ICA was applied where other artifacts including eye movements, eye blinks, and muscle artifacts were removed. 

In order to avoid the effect of skull-induced volume conduction on the precision of phase estimation, a surface Laplacian algorithm, current source density (CSD), was used. In this regard, the signal is transformed into the CSD domain [30].

### 2.5. EEG Post-Processing

**Figure 1:**
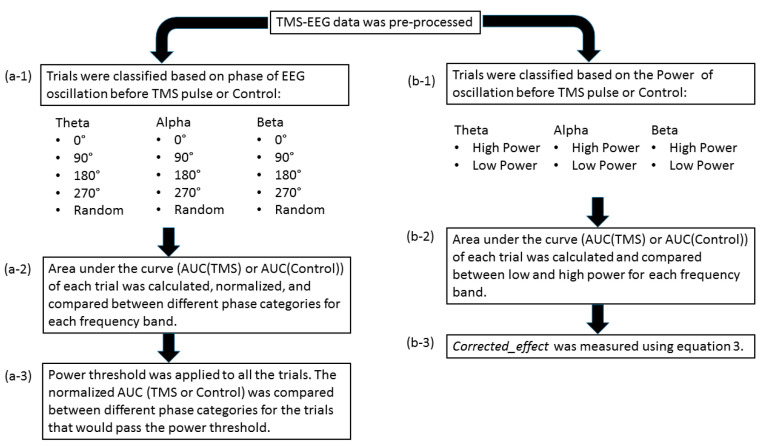
Overall post-processing analysis flow: (**a-1**) the trials were classified based on the phase of the EEG oscillation before the TMS pulse or control condition using PHASTIMATE toolbox; (**a-2**) the single-trial TMS-EEG-evoked response, measured by area under the curve of the waveform between 25 and 80 ms (after the TMS pulse or the time of control condition), was compared between different phase categories in each frequency band; (**a-3**) the mean of power for each subject and every frequency was set as the threshold, and then the AUC of the trials that would pass the threshold were compared between different phase categories in each frequency band; (**b-1**) the trials were classified based on the power of the EEG oscillation before TMS pulse or control condition into high power (above the median—comprising 50% of the data) and low power (below the median) in each frequency band; (**b-2**) AUC of high power and low power were compared in each frequency band; (**b-3**) to isolate the effect of TMS from ongoing brain oscillation, *corrected_effect* was measured.

The post-processing steps included categorizing the trials based on the phase and power of EEG oscillations before TMS pulse and comparing the single-trial TMS-EEG response, measured by the area under the curve, in different phase/power categories (please see Figure 1). The same procedure was applied for the control condition in the data. The detailed explanation of each step is presented in the following.

**Figure 2:**
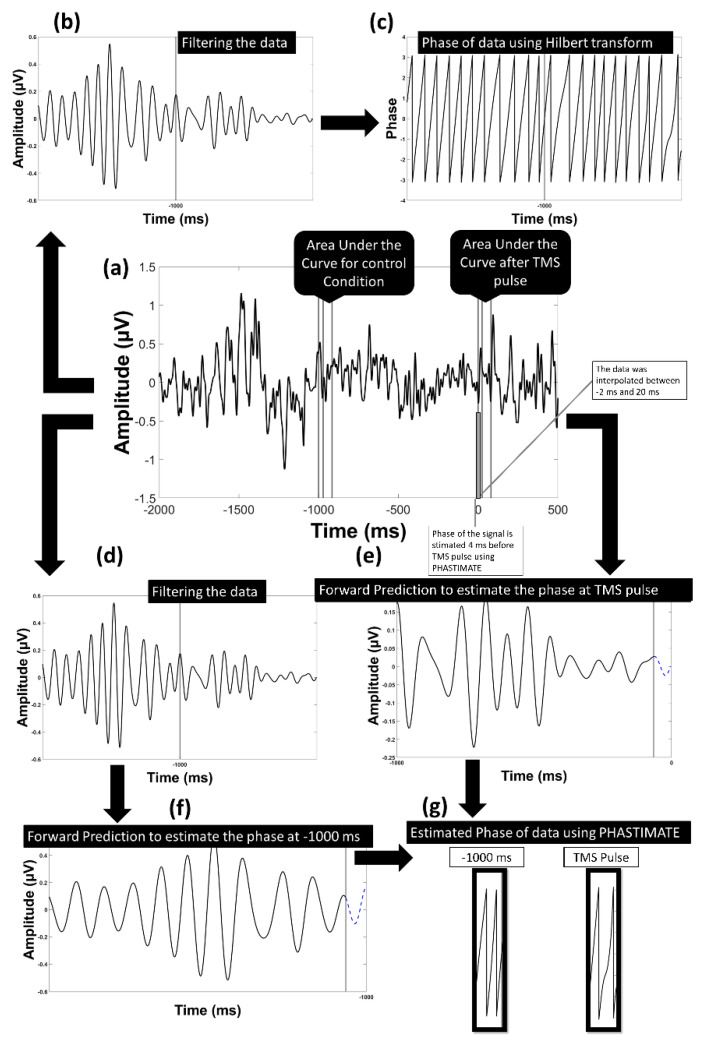
Model of the analysis flow to estimate the phase in the alpha band: (**a**) the data transformed into current source density domain; (**b**) the data filtered into alpha frequency band; (**c**) the phase of the filtered data using Hilbert transform was estimated; (**d**) the data filtered into alpha frequency band to be used in PHASTIMATE toolbox; (**e**) forward prediction of the signal before TMS pulse after edging it using PHASTIMATE toolbox; (**f**) forward prediction of the signal 1000 ms before TMS (control condition) after edging it using PHASTIMATE toolbox; (**g**) the estimated phase of the signal using PHASTIMATE toolbox and Hilbert transform.

**Figure 3:**
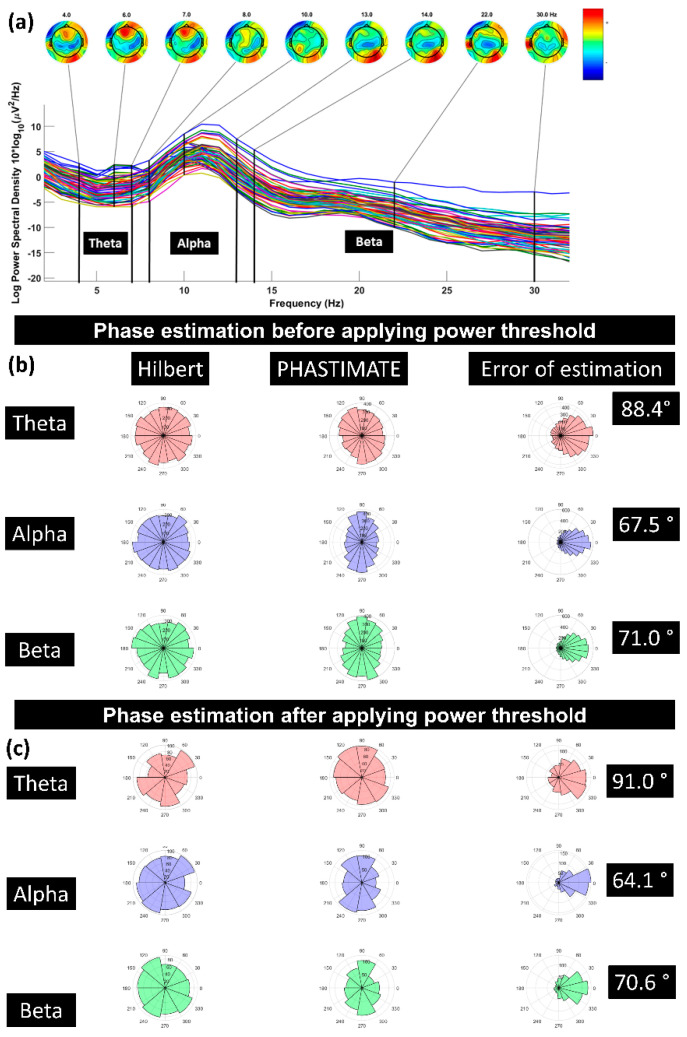
(**a**) A sample power spectrum for the frequency bands of theta (4–7 Hz), alpha (8–13 Hz), and beta (14–30 Hz); (**b**) error of phase estimation, using PHASTIMATE toolbox (second column) compared with Hilbert transform (first column) in all the trials, in each frequency band, and without applying the power threshold; (**c**) error of phase estimation in all the trials which passed the power threshold (mean of power in each frequency band and for every subject). Error of estimation was measured using circular standard deviation [31]. The phase in all the plots is estimated for 1000 ms before TMS pulse.

The single trials were analyzed for the F3 electrode. The electrode F3 was chosen as the electrode of interest since it is the closest electrode to the stimulation site (DLPFC). We used the signal recorded from F3 after re-referencing to a common average reference. The PHASTIMATE algorithm [31] was used to estimate phase at the time of the stimulation based on pre-stimulus data only. This algorithm has been used in various real-time EEG-triggered TMS experiments [15,32] and is available as an open-source MATLAB toolbox (github.com/bnplab/phastimate). It has also recently been compared to other algorithms [33] and has been shown to perform comparatively well in conditions of high noise. Instantaneous phase was estimated for the theta (4–7 Hz), alpha (8–13 Hz), and beta (14–30 Hz) frequency bands. PHASTIMATE parameters were set based on Table 1, selected according to previous studies [31,32], and adjusted to minimize the error of estimation. The PHASTIMATE algorithm parameters were optimized for a minimum error at an arbitrary time point of −1000 ms (before TMS) between the two TMS pulses where no effect of previous pulse and upcoming pulse is available. For this, the Hilbert transform was used to estimate the real phase and adjust the parameters of the phase estimation algorithm to ensure minimum error for each frequency band (Figure 2 and Figure 3). To remove the TMS artifact, data between the time points of −2 ms and 20 ms were removed and linearly interpolated. Therefore, the last time point available for phase estimation based on PHASTIMATE was −4 ms, which also avoided the ripple and edge effect of the anti-aliasing filter (Figure 2). 

### 2.6. Single-Trial Response to TMS Pulse 

We measured the area under the curve (AUC(TMS)) of single trials (Equation (1)) from the recorded waveform resulting from a single TMS pulse. The AUC(TMS) represents the sum of all activity over the time window, with greater AUC(TMS) indicating a larger response to the TMS pulse. This is not equivalent to the more commonly reported TMS-evoked potential (TEP) which represents the average of all single-trial responses (please see Figure S1 illustrating the TEP). The AUC(TMS) was computed for the time window of 25 ms to 80 ms. This time window was selected as the signal is shown to be free from somatosensory and auditory artifacts [34,35,36,37]. Equation (1):

(1) $$\mathrm{AUC}\left(\mathrm{TMS}\right)=\int_{25\;\mathrm{ms}}^{80\;\mathrm{ms}}\left|Single\;Trial\;Response\right|$$

Since AUC(TMS) contains information regarding both excitatory and inhibitory mechanisms (i.e., both peaks and troughs in the defined time period) [23], it was considered as the main measure to compare the recorded responses of various phases. It should be noted that by choosing AUC as the measure of cortical activity, the information regarding the amplitude of deflections in the waveform will not be considered as a measure, therefore no conclusion can be drawn regarding the underlying excitatory or inhibitory mechanisms of the waveforms. Moreover, AUC may have limited sensitivity to the type of change in the evoked response where peak magnitudes change in opposite directions (some peaks increasing, others decreasing). Five categories of Positive Peak (0 ± 30°), 90° (±30°), Negative Peak (180 ± 30°), 270° (±30°), and Random Phase (0 ± 180°, i.e., all of the trials) were defined based on the phase of the signal before TMS pulse. The trials and their subsequent AUCs were categorized based on these five categories for every participant. Statistical analysis was performed for each participant as follows: Trials were sorted across phase category (i.e., 0° (±30°), 90° (±30°), 180°(±30°), 270° (±30°)), and the average AUC was determined for each of the four subsets of trials. The average AUC across all of the trials of that participant (i.e., random phase) was then subtracted, yielding an average difference metric (normalized AUC) for each of the four phase conditions for each participant. Then, across the participant averages, two-sided paired *t*-tests were performed to compare the normalized AUC of each phase category and over the theta, alpha, and beta frequency bands. The significance level was adjusted (0.004) to account for the 12 (i.e., number of frequencies*number of phases) multiple comparisons (0.05/12).

### 2.7. The Control Condition: Single-Trial Response without TMS Pulse

As the control condition, we explored whether there is an effect of the phase of ongoing brain oscillation on the EEG signal without the presence of a TMS pulse. To do so, we calculated the area under the curve (will be called AUC(Control) throughout the manuscript) of the signal for the time window of −971 ms to −916 ms (the same length of the time window as of after TMS pulse (Equation (2)), categorized the trials based on the phase of the signal at −1000 ms (to be consistent with the TMS condition, the phase was estimated for −1000 ms and the area under the curve for 29 ms after that). The same method of normalization and statistical tests as AUC(TMS) were applied for the AUC(Control). The phase of the signal at −1000 ms and the subsequent AUC(Control) were chosen as the control condition since there is no effect of TMS pulse on the signal, either the pulse from the previous trial or the upcoming one. An earlier time is not possible as it may then overlap with the evoked potential following the preceding TMS pulse. Equation (2):

(2) $$\mathrm{AUC}\left(\mathrm{Control}\right)=\int_{-971\;\mathrm{ms}}^{-916\;\mathrm{ms}}\left|Single\;Trial\;Response\right|$$

### 2.8. Power Threshold to Exclude Trials with Low Power

As an additional step in exploring the effect of phase of ongoing brain oscillation on the single-trial TMS-EEG response, we applied a power threshold to exclude the trials with low power. The power threshold was defined as the average of the power of the signal in each frequency band and for every subject. Therefore, the statistical analysis to compare the AUC(TMS) in different phase categories was performed for the trials that passed the power threshold as well (Table 2). The same procedure of applying power threshold was performed for AUC(Control) in different phase categories. 

The statistical analysis was performed using MATLAB (R2018b), and the boxplots using IoSR Matlab Toolbox (github.com/IoSR-Surrey/MatlabToolbox (accessed on 12 August 2019).).

### 2.9. Power Spectrum Calculation

Power at each frequency band was calculated using Welch’s method for the time window of 1000 ms in theta, 500 ms in alpha, and 250 ms in beta bands before the TMS pulse/time of the control window. To explore the correlation between the power of the signal in each frequency band and AUC(TMS), the trials were divided into two bins: low power (below the median—comprising 50% of the data) and high power (above the median). For every subject, the average of AUC(TMS) was computed for low-power trials and high-power trails. Two-sided paired Student’s *t*-tests were then performed to compare the AUC(TMS) between low and high power and for each frequency band. The same procedure was applied for the control conditions. The significance level was set at 0.017 (*p*-value < 0.05/3 (3 frequencies)) to correct for multiple comparisons. 

A major challenge of this analysis is that the resulting post-stimulus EEG signal is a combination of two effects: the effect of the TMS pulse on the post-stimulus EEG and the effect of the pre-stimulus ongoing activity on the post-stimulus EEG (i.e., if power was high before the TMS pulse, it is likely high after the TMS pulse). We attempt to disentangle the contribution of these two effects by quantifying the interaction of the TMS pulse with the EEG signal given the pre-stimulus power by performing an additional correction in our analysis.

**Figure 4:**
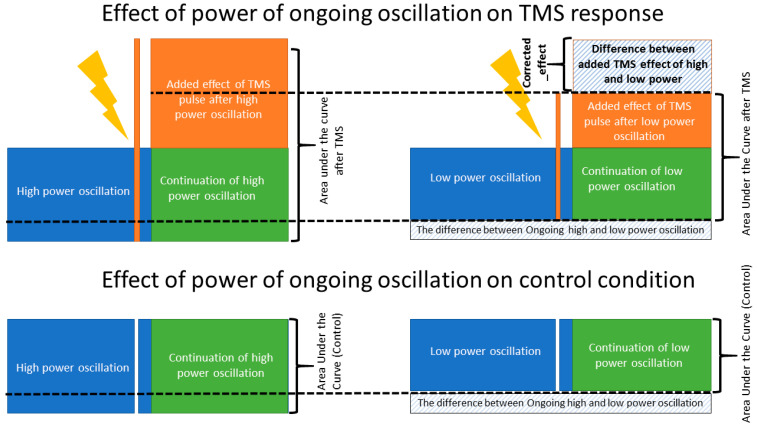
*Corrected_effect*. A schematic figure, illustrating the effect of ongoing brain oscillation on the post stimuli area under the curve and the added evoked response of TMS to the amplitude of ongoing low- and high-power oscillations. The *corrected_effect*, calculated based on Equation (3), illustrates the isolated effect of TMS pulse when the effect of ongoing brain oscillation is removed. The *corrected_effect* was significantly larger than zero only in the theta and beta bands (*p* < 0.01).

EEG data is temporally correlated, meaning that signal properties (oscillatory power) at time 0 predicts signal properties at time +t [38,39,40]. We therefore used control segments of data between TMS pulses to quantify the influence of pre-stimulus EEG on our post-stimulus AUC measure in the absence of a stimulus. To disentangle the effect of pre-stimulus power due to ongoing EEG dynamics from any effect of cortical reactivity to TMS, we then subtracted the effect observed in the non-TMS control condition. Specifically, in every frequency band, the AUC(TMS) in the low power condition was subtracted from AUC(TMS) in the high-power condition. Similarly, the AUC(Control) in the low power condition was subtracted from the AUC(Control) in the high-power condition. Then, a measure named corrected_effect was defined as the outcome of the subtraction of low from high power AUC in TMS minus the outcome of the subtraction of low from high power AUC in the control condition (please see Figure 4 and Equation (3)). Then, a two-sided paired *t*-test was performed to check if corrected_effect was significantly different from zero which would identify the presence of a significant TMS effect in addition to the influence of the ongoing EEG oscillation. The significance level was set at 0.017 (*p*-value < 0.05/3 (3 frequencies)) to correct for multiple comparisons. Equation (3):

(3) $$Corrected\_effect=\left(\mathrm{AUC}\left(High\_power_{TMS}\right)-\mathrm{AUC}\left(Low\_power_{TMS}\right)\right)-\;\left(\mathrm{AUC}\left(High\_power_{Control}\right)-\;\mathrm{AUC}\left(Low\_power_{Control}\right)\right)$$

## 3. Results

### 3.1. Effect of Pre-TMS Phase of Ongoing Brain Oscillation on TMS Cortical Response

**Figure 5:**
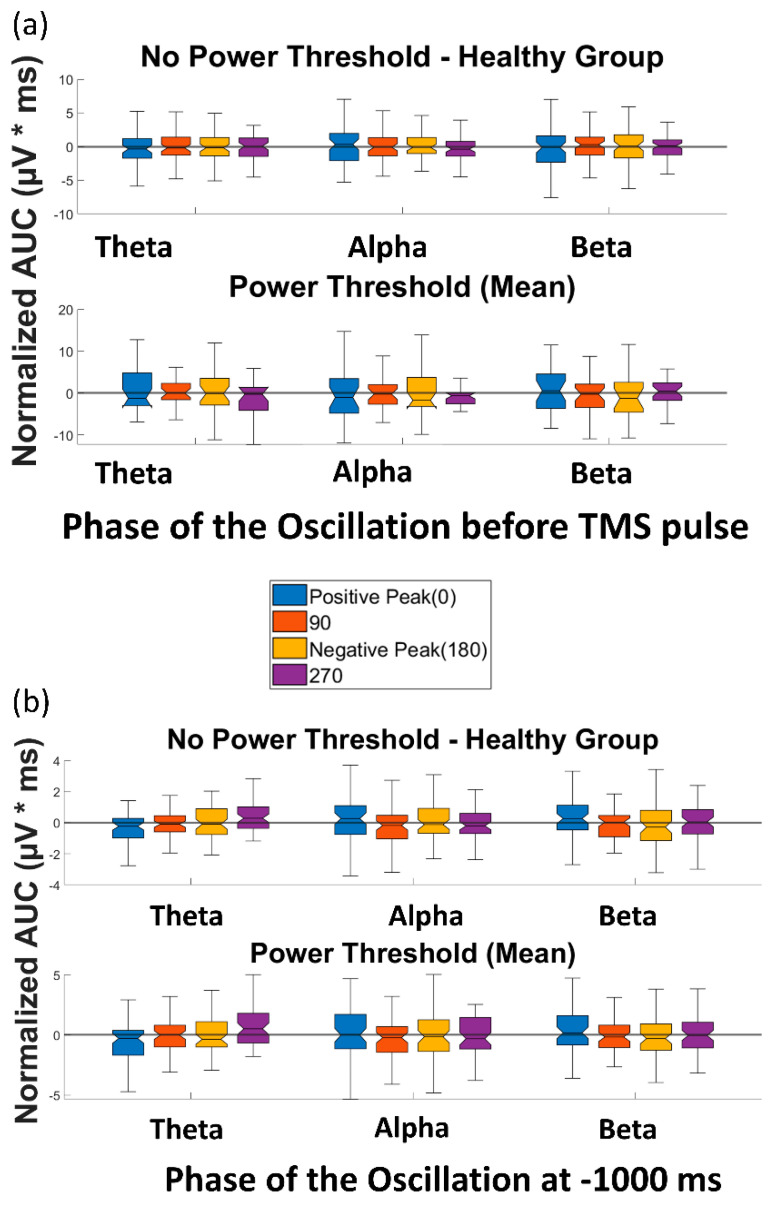
Effect of phase of ongoing EEG oscillation on single-trial TMS-EEG response. (**a**) Mean rectified TMS-evoked EEG activity, measured by normalized AUC_(TMS)_, for different phases of positive peak, 90°, negative peak, and 270° over the three frequency bands of theta, alpha, and beta before and after the power threshold (mean power) is applied. (**b**) Comparison of the normalized area under the curve for the control condition (AUC_(Control)_) for different phases of positive peak, 90°, negative peak, and 270° over the three frequency bands of theta, alpha, and beta before and after the power threshold (mean power) is applied. None of the comparisons reached the significance level.

The four paired *t*-tests comparing AUC(TMS) for each of the four phases with AUC(TMS) for the random phase revealed no significant differences over any of the frequency bands of theta, alpha, and beta (please see Table S1 for detailed *p*-values). When a power threshold was applied to exclude low-power trials, no significant difference was observed between the AUC(TMS) of different phases or over the three aforementioned frequency bands (Figure 5a). Therefore, our analysis did not demonstrate a modulation of single-trial TMS reactivity by the phase of ongoing brain oscillation in the datasets used in this study.

### 3.2. Effect of Phase of Ongoing Brain Oscillation in Control Condition

Similarly, the *t*-tests to compare the AUC(Control) of different phase categories in the control condition (without TMS) revealed no significant difference between the AUC(Control) of different phases or over any of the frequency bands of theta, alpha, and beta (please see Table S1 for detailed *p*-values). When the power threshold was applied to the trials, no significant difference was observed between the AUC(Control) of different phases and over the three aforementioned frequency bands (Figure 5b). Therefore, as expected, no effect of phase was observed in the control condition of the datasets in this study.

### 3.3. Power of the Signal and the Recorded Response

**Figure 6:**
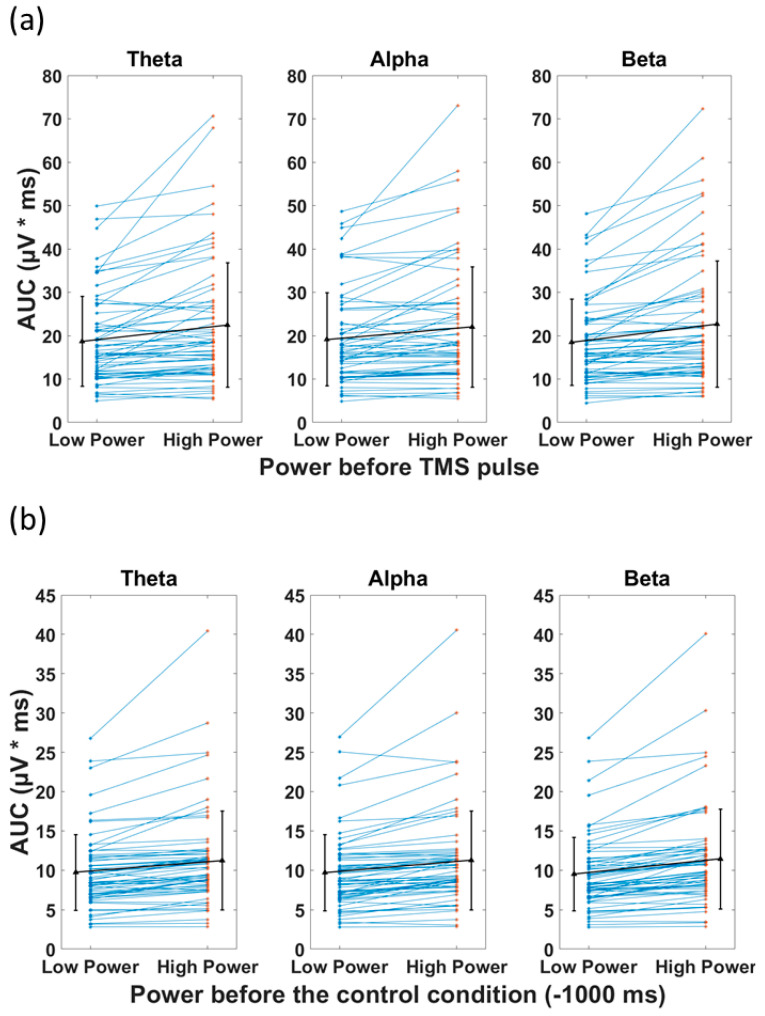
Effect of power of ongoing EEG oscillation on single-trial TMS-EEG response measured by area under the curve (AUC) after TMS pulse and in the Control condition with mean and standard deviation also shown (black). (**a**) Mean rectified TMS-evoked EEG activity, measured by area under the curve (AUC_(TMS)_) for low power and high power over the three frequency bands of theta, alpha, and beta in healthy group. (**b**) Comparison of the area under the curve for the control condition (AUC_(Control)_) for low power and high power over the three frequency bands of theta, alpha, and beta. High power resulted in significantly larger AUC (*p* < 0.001) compared with low power, both in TMS and control conditions in theta, alpha, and beta frequency bands.

The *t*-tests comparing the AUC (TMS), categorized based on pre-stimulus low power and high power, revealed a significant difference between the two categories for all frequency bands (Figure 6a). The high power resulted in significantly larger AUC(TMS) compared with low power in the theta (22.5 (mean) ± 14.46 (SD) µV*ms vs. 18.7 ± 10.3 µV*ms, *p* < 0.001), alpha (22.0 ± 13.9 µV*ms vs. 19.1 ± 10.7 µV*ms, *p* < 0.001), and beta (22.7 ± 14.6 µV*ms vs. 18.5 ± 9.9 µV*ms, *p* < 0.001) bands. In the control condition, the *t*-tests revealed significant difference between AUC(Control) of low and high power (Figure 6b). The high power resulted in significantly larger AUC(Control) compared with low power in the theta (11.2 ± 6.3 µV*ms vs. 9.7 ± 4.8 µV*ms, *p* < 0.001), alpha (11.3 ± 6.3 µV*ms vs. 9.7 ± 4.8 µV*ms, *p* < 0.001), and beta (11.4 ± 6.4 µV*ms vs. 9.5 ± 4.7 µV*ms, *p* < 0.001) bands (please see Table 3 for detailed statistics). The associations of the preceding power with AUC(TMS) and AUC(Control) had the same direction. This observed phenomenon can be explained as the continuation of the EEG power (before the pulse) after the TMS pulse. 

The two-sided *t*-tests were performed to compare whether corrected_effect would be significantly different from zero. Corrected_effect was significant in the theta (2.3 ± 6.2 µV*ms, t(63) = 2.9, *p* < 0.01) and beta (2.3 ± 6.2 µV*ms, t(63) = 2.9, *p* < 0.01) bands, but not in the alpha (1.3 ± 5.9 µV*ms, t(63) = 1.7, *p* = 0.085) band. This suggests that the effect of power of the signal before TMS pulse, though also observed in the control condition, cannot be purely explained by the ongoing high versus low amplitude activity in the theta and beta bands.

## 4. Discussion

### 4.1. Summary

In this study, we investigated whether the characteristic features of ongoing brain oscillations (i.e., phase and power) at the time of stimulation have any impact on the single-trial evoked EEG response to TMS over the left DLPFC in a relatively large dataset of healthy participants. We observed that a higher power across all frequency bands prior to the TMS pulse was associated with a larger AUC of the recorded waveform resulting from a single TMS pulse. However, a significant effect of the power of the signal was also observed in the control condition (without a TMS pulse), which is trivially explained by a continuation of the ongoing dynamics from the preceding period to the interval of AUC computation. After correcting for the continuation of pre-stimulus oscillation (*corrected_effect*; Equation (3)) we also observed that cortical reactivity to TMS (AUC) is larger during high-power trials compared to low-power trials (in theta and beta, but not in alpha). With regard to phase, while the pre-stimulus phase was previously found to affect the amplitude of evoked EMG potentials following TMS over M1, here, no effect was observed for the phase of pre-stimulus EEG on single-trial TMS-evoked EEG responses.

### 4.2. Phase and Cortical Response

Given the previous findings, an instantaneous phase of an ongoing brain oscillation is expected to be related to cortical reactivity [41,42,43]. Multiple studies have demonstrated that the amplitude of evoked EMG responses, as an index of corticospinal excitability, is modulated by the phase of pre-stimulus EEG [15,44,45,46,47] or EMG [19]. However, even in the motor cortex, where the modulatory effect of phase on EMG response is observed, it has so far not been possible to demonstrate a similar effect in TMS-EEG outcome measures in an offline setting [17]. The nil effect of phase of the signal on AUC(TMS), observed in our study over DLPFC, needs to be considered in the context of the similar nil effect in the primary motor cortex [17], where the alpha-phase modulation is known to play a role. 

Why has it been difficult to demonstrate an effect of pre-stimulus phase in TMS-EEG? There are notable differences between an EMG index of cortical excitability used in the aforementioned studies and the EEG AUC index used in our study. Those studies examined the effect of phase on the EMG response to TMS delivered to the motor cortex, whereas we examined TMS-EEG following TMS to the DLPFC. It is important to keep in mind that the mechanism of the recorded response in EMG and EEG are different and each reflect different aspects of cortical activation [17]. While the MEP response reflects the cortico-spinal excitability in response to TMS [1,48], the EEG signal is the product of the summation of both excitatory and inhibitory cortical activations occurring at the macroscopic level [17]. However, while EMG amplitudes are a very indirect measure of cortical excitability, being affected by spinal processes and muscular activities, the EEG signal is a direct reflection of neurophysiological activity at the cortex which also makes it more difficult to control for experimental variables and interpret the results [5]. In addition to the differences between EEG and EMG, the difference between the cytoarchitecture of DLPFC and the motor cortex should also be taken into consideration [49]. Accordingly, the possible modulatory effect of phase of ongoing brain oscillations, observed in MEP responses, cannot necessarily be reflected in the EEG signal of DLPFC. 

Importantly, as discussed by [45], the number of trials for each condition must exceed the minimum of 100 to be able to observe the modulatory effect of the phase of the EEG signal oscillation. Although in our study we tried to compensate for the relatively low number of trials by combining the trials for each subject together, it cannot be overlooked that the trials accumulated for each phase were not recorded from the same participant. According to [45], in order to observe the effect of phase based on the five categories that we have considered (positive peak, 90°, negative peak, 270°, and random) at least 500 pulses must be delivered (100 per condition) to perform a direct comparison. However, this limitation in our study provides insight toward the usual TMS-EEG studies. Since the majority of TMS-EEG studies deliver 200 pulses or less [3] in neurophysiology research, the effect that phase might have (and was not detected in our study) on the variability of the response in TMS-EEG response is not significant. Finally, the inter-trial interval was also discussed by [18] as a possible source based on which the modulatory effect of the phase of the EEG signal can be determined. In summary, further developments in experimental procedures and analysis methods may be required in order to investigate the modulatory effect of the phase of EEG oscillations on the recorded response. Specifically, this includes a significantly larger number of trials per participant and improved procedures to reduce TMS-related artifacts and sensory co-activation, as well as analytic methods that separate the contribution of ongoing oscillations from evoked responses.

### 4.3. Power and Cortical Response

A significant effect of pre-stimulus power on DLPFC reactivity to TMS was found in theta and beta frequency bands (but not alpha), with high power pre-stimulus trials resulting in a larger evoked response in terms of AUC after correcting for the effect of ongoing dynamics. It has previously been shown [20,44,47] that sensorimotor alpha and beta EEG power before the TMS pulse can have a modulatory effect on MEP amplitude when TMS is applied to the motor cortex. However, not every study reports that high power is associated with higher cortical reactivity. In fact, it is generally assumed that high power is a result of the synchronization of neuronal activity which reflects a state of low responsivity to external drivers [50,51]. For alpha, high power was shown to correspond to low excitability in the occipital region [52,53] and the sensorimotor cortex [54,55]. The same negative association was observed for beta over the motor cortex [44,56]. No study has so far investigated the effect of theta power on MEP or EEG outcome measures of TMS in human participants to the best of our knowledge. However, even for the most frequently investigated alpha frequency band, no simple relationship between power and cortical excitability was found [57,58], but higher alpha power is also reported to result in larger sensorimotor cortex excitability as indexed by MEP amplitude [59], similar to our finding for theta and beta in DLPFC.

Why did we see this effect in the theta and beta frequencies, but not in the alpha band? Our study investigated single-trial TMS reactivity in the frontal cortex. Cognitive control was shown to be reflected in frontal theta oscillation [60]. In a single-trial analysis, [61] demonstrated the role of frontal theta in cognitive control as well. Power of theta in the frontal cortex is linked to working memory performance [62]. Increase in theta power is also associated with cognitive conflict [63,64] and error [65,66]. Moreover, the power of the theta and beta bands in the prefrontal cortex has also been associated with cognitive performance [67]. On the other hand, although frontal alpha asymmetry was thought to be a predictive biomarker in depressive disorder [68,69], this was not borne out by recent studies [70]. According to the studies mentioned in this paragraph, the theta and beta bands are seemingly the biophysical realization of higher functions in the prefrontal cortex.

In our study, TMS evoked higher levels of cortical activity when the pulses were delivered at high power theta and beta frequencies compared with lower power. This effect is worth exploring further in a prospective study as it can provide insight towards decreasing the TMS-EEG variability of the response as well as increasing the effectiveness of the treatment considering that DLPFC is the main treatment target for MDD. The feasibility [32] and modulatory effect (for phase) [71] of theta-synchronized TMS over the frontal area of the cortex is successfully performed. Based on the findings of the current study and the evidence highlighting the role of frontal theta oscillations in psychiatric disorders [72], power in the theta frequency band can be a candidate to synchronize TMS pulses with endogenous brain rhythms based on the oscillatory features of EEG signal.

### 4.4. Variability of Cortical Reactivity in TMS-EEG

The variability of results in TMS-EEG studies is relatively high and has been a source of concern [41,73,74]. A part of this variability can be explained by differences in devices and methods of determining TMS intensity, as well as pre-processing and analysis approaches [74]. The standard operating procedures include appropriate noise masking and coil-scalp spacing to limit the sources of variability [37]. The effect of the phase of beta oscillations before the TMS pulse (over superior parietal lobe) on inter-trial variation in TMS-evoked potentials, measured by global mean field amplitude, was demonstrated by [75]. Here, we were not able to observe the role that the phase of the ongoing brain oscillation can play in this variability and found the modulatory effect of the power of theta and beta oscillation before TMS pulse. To have a better understanding of the effect of the features of ongoing brain oscillations on the variability of response in TMS-EEG studies, EEG-triggered TMS is needed in future studies to test the effect of the power and phase of the endogenous brain oscillations before the TMS pulse on single-trial TMS-EEG responses. Moreover, standardization of the recording of TMS-EEG signals and sharing of data in public repositories is essential as it can help researchers across different laboratories to access more data to perform new analytical approaches with the advancements in analysis methods.

### 4.5. Limitations

We would like to discuss the following limitations: First, we used standard F3 vs. avg. reference to extract local oscillatory activity, as opposed to spatial filters optimized to extract the relevant frontal frequencies (e.g., using spectro-spatial decomposition, beamforming, etc.). While we acknowledge that this approach may not be the optimal montage to extract the signal of interest, we consider an evaluation of alternative EEG transforms to be beyond the scope of this study. Partly as a result of this, even though we have used a state-of-the-art method of phase estimation, we have nevertheless observed considerable error in the estimation of phase. Second, the number of pulses in our study for each participant was limited compared with the number of pulses delivered in studies which have reported a positive effect of phase. As an example, [31] have introduced a sample dataset with 1200 pulses for only one subject which provides a great opportunity to investigate the effect of phase of the signal, meaning that by dividing the signal into various phase categories, significantly larger numbers of trials from the same participant will be available to increase the likelihood of observing the effect of the phase of the signal on TMS response. Moreover, we could not perform within subject comparisons of the trials due to the limited number of trials for each participant. Third, given that these data were collected as part of earlier studies, some of our data were collected prior to current standards. TMS-EEG standards of data collection have evolved considerably over recent years and the state-of-the-art protocols (application of a layer of foam and noise masking) may help to improve data quality and subsequently produce more reliable results. Fourth, it is not possible to disentangle all the concurrent physiological brain frequencies which might also interact with each other. These can have effects that are not dealt with in the framework of this work. In addition, in our analysis we dichotomized the phases into bins in order to make our study comparable with the studies performed previously. Whether the shape of the magnetic pulse (monophasic vs. biphasic) has any interactive modulatory effect with the EEG oscillatory features on the outcomes of the stimulation is a topic worth exploring but it is beyond the scope of the current work. In this study, we mainly focused on the response in the early time window of below 80 ms as there have been several studies both investigating the underlying mechanisms of the TEP waveform and the reliability of it in this time window. However, as evidenced by some studies [36,76], the cortical activity after TMS beyond 400 ms can also be explored to investigate the possible relationship between pre-TMS phase and power with cortical reactivity to TMS. Every *t*-test was performed on 64 values obtained from 49 subjects, with 15 individuals contributing 2 values each. Thus, the assumption of independent measurements was violated, and the tests may have produced incorrect significance levels. We further performed the same analysis on 49 datasets by including the best available dataset between the two recordings (recordings with the state-of-the art masking procedure for sensory co-activation). The significant and non-significant findings of the manuscript were replicated in this further step of analysis. Finally, our main result depends on a correction method that is based on assumptions which may not be fully met; specifically, it assumes linearity in the interaction between the TMS pulse and the ongoing EEG oscillation.

### 4.6. Conclusions

Brain-state-dependent brain stimulation needs to be based on a complete picture regarding how various features of EEG oscillations before the stimulus, over various parts of the cortex, influence brain responses to stimulation. Our study provides a step towards completing this picture through the observation that the power of EEG oscillations in theta and beta bands prior to the TMS pulse is associated with cortical excitability over DLPFC. This is one step toward personalized medicine based on each individual’s unique endogenous neural activity. As this study implemented a retrospective design, it warrants further investigation in a prospective setting to explore the effect of EEG oscillations on the TMS-EEG signal as well as the therapeutic application of oscillation-synchronized rTMS. 

## Figures and Tables

**Table 1:** The parameters for PHASTIMATE toolbox used in this study for theta, alpha, and beta bands.

Frequency	Filter Order (Zero-Phase Forward and Backward)	Window Size	Removed Edge	Order (Yule–Walker)	Hilbert Window
Theta (4–7 Hz)	320	1000 ms	140 ms	30	256
Alpha (8–13 Hz)	192	718 ms	65 ms	25	128
Beta (14–30 Hz)	100	500 ms	30 ms	15	64

**Table 2:** Total number of trials in each phase category before and after applying the power threshold (mean of power for each frequency band and every subject).

No Power Threshold					
**Frequency/Phase**	**Random**	**Positive Peak**	**90°**	**Negative Peak**	**270°**
Theta	5347	799	999	722	1045
Alpha	5347	658	1264	673	1121
Beta	5347	670	1125	642	1172
**Power Threshold Applied**					
**Frequency/Phase**	**Random**	**Positive Peak**	**90°**	**Negative Peak**	**270°**
Theta	660	109	127	100	123
Alpha	732	80	168	88	153
Beta	683	85	152	82	145

**Table 3:** Student’s *t*-test results of comparing the area under the curve (AUC_(TMS)_ or AUC_(Control)_) based on the pre-stimulus power (low power vs. high power) over theta, alpha, and beta frequency bands. Significance level (*p*) was adjusted for multiple comparison as 0.008 (0.05/6). Std = standard deviation, low power = below the median, high power = above the median, df = degree of freedom.

*t*-Test Statistics Comparing the Area under the Curve of TMS for Low vs. High Pre-TMS Power in Healthy Group							
**Frequency**	**Mean-Low**	**Std-Low**	**Mean-High**	**Std-High**	**t.stat**	**df**	***p***
Theta	18.7	10.3	22.5	14.4	4.7	63	1.27 × 10^−5^
Alpha	19.1	10.7	22.0	13.9	4.2	63	8.67 × 10^−5^
Beta	18.5	9.9	22.7	14.6	5.4	63	1.01 × 10^−6^
***t*-Test Statistics Comparing the Area under the Curve of Control Condition for Low vs. High Power in Healthy Group**							
Theta	9.7	4.8	11.2	6.3	5.7	63	3.67 × 10^−7^
Alpha	9.7	4.8	11.3	6.3	5.6	63	4.39 × 10^−7^
Beta	9.5	4.7	11.4	6.4	6.7	63	7.07 × 10^−9^

## Data Availability

The datasets used during the current study are available by contacting Daniel Blumberger (daniel.blumberger@camh.ca) on reasonable request.

## Supplementary Materials

The following supporting information can be downloaded at: https://www.mdpi.com/article/10.3390/bios13020220/s1, Figure S1: TMS-evoked potential (TEP). TEP measured by the average of all trials for 64 datasets. The red line shows the recording from electrode F3; Table S1: *p*-values as the result of *t*-tests comparing the normalized area under the curve (AUC) of each phase bin with random in TMS and Control conditions.
