# Supplementary material for: Pre-Stimulus Power but Not Phase Predicts Prefrontal Cortical Excitability in TMS-EEG
Source: Biosensors (Basel). 2023 Feb 3;13(2):220. doi: 10.3390/bios13020220 (PMC9953459; doi:10.3390/bios13020220)
Supplement: Supplementary file 1 [file biosensors-13-00220-s001.zip › biosensors-1983092-supplementary.pdf]

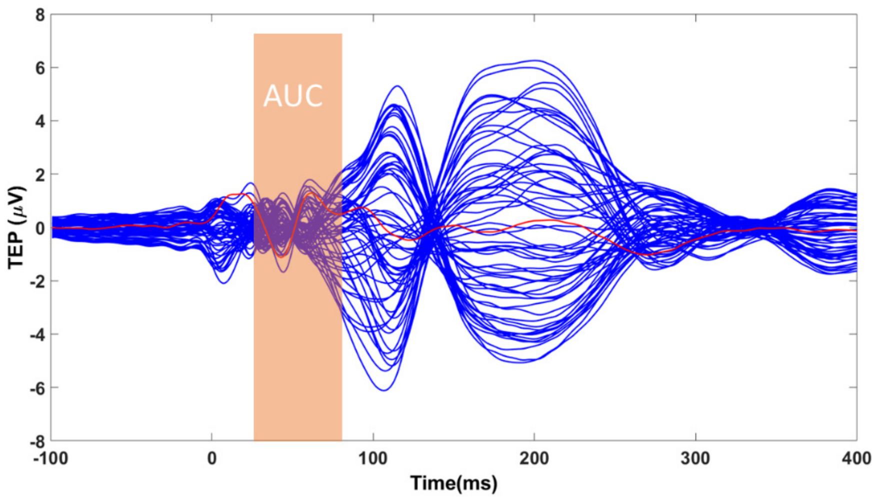

Figure S1. TMS-evoked potential (TEP). TEP measured by the average of all trials for 64 datasets. The red line shows the recording from electrode F3.

	P-values comparing normalized AUC of each phase bin with random phase			
	Pre-TMS			
	0°	90°	180°	270°
Theta	0.23	0.85	0.50	0.73
Alpha	0.59	0.85	0.74	0.68
Beta	0.46	0.91	0.47	0.20
	Control			
	0°	90°	180°	270°
Theta	0.05	0.60	0.80	0.03
Alpha	0.37	0.34	0.40	0.96
Beta	0.59	0.23	0.78	0.29

Table S1. P-values as the result of t-tests comparing the normalized area under the curve (AUC) of each phase bin with random in TMS and Control conditions.
